# ATF3 contributes to brucine-triggered glioma cell ferroptosis via promotion of hydrogen peroxide and iron

**DOI:** 10.1038/s41401-021-00700-w

**Published:** 2021-06-10

**Authors:** Shan Lu, Xuan-zhong Wang, Chuan He, Lei Wang, Shi-peng Liang, Chong-cheng Wang, Chen Li, Tian-fei Luo, Chun-sheng Feng, Zhen-chuan Wang, Guang-fan Chi, Peng-fei Ge

**Affiliations:** 1 Department of Neurosurgery, First Hospital of Jilin University, Changchun, 130021 China; 2 Research Center of Neuroscience, First Hospital of Jilin University, Changchun, 130021 China; 3 Department of Neurology, First Hospital of Jilin University, Changchun, 130021 China; 4 Department of Anesthesiology, First Hospital of Jilin University, Changchun, 130021 China; 5 Key Laboratory of Pathobiology, Ministry of Education, Jilin University, Changchun, 130021 China

**Keywords:** glioma, brucine, ferroptosis, ATF3, hydrogen peroxide, ER stress, NOX4

## Abstract

Ferroptotic cell death is characterized by iron-dependent lipid peroxidation that is initiated by ferrous iron and H_2_O_2_ via Fenton reaction, in which the role of activating transcription factor 3 (ATF3) remains elusive. Brucine is a weak alkaline indole alkaloid extracted from the seeds of *Strychnos nux-vomica*, which has shown potent antitumor activity against various tumors, including glioma. In this study, we showed that brucine inhibited glioma cell growth in vitro and in vivo, which was paralleled by nuclear translocation of ATF3, lipid peroxidation, and increases of iron and H_2_O_2_. Furthermore, brucine-induced lipid peroxidation was inhibited or exacerbated when intracellular iron was chelated by deferoxamine (500 μM) or improved by ferric ammonium citrate (500 μM). Suppression of lipid peroxidation with lipophilic antioxidants ferrostatin-1 (50 μM) or liproxstatin-1 (30 μM) rescued brucine-induced glioma cell death. Moreover, knockdown of ATF3 prevented brucine-induced accumulation of iron and H_2_O_2_ and glioma cell death. We revealed that brucine induced ATF3 upregulation and translocation into nuclei via activation of ER stress. ATF3 promoted brucine-induced H_2_O_2_ accumulation via upregulating NOX4 and SOD1 to generate H_2_O_2_ on one hand, and downregulating catalase and xCT to prevent H_2_O_2_ degradation on the other hand. H_2_O_2_ then contributed to brucine-triggered iron increase and transferrin receptor upregulation, as well as lipid peroxidation. This was further verified by treating glioma cells with exogenous H_2_O_2_ alone. Moreover, H_2_O_2_ reversely exacerbated brucine-induced ER stress. Taken together, ATF3 contributes to brucine-induced glioma cell ferroptosis via increasing H_2_O_2_ and iron.

## Introduction

Gliomas are the most common type of malignant brain tumors with higher mortality. The median survival of the patients with high-grade glioma is not longer than 14.6 months, even if they accept surgery and then are treated with chemotherapy and radiotherapy [1]. It has been found that glioma cells are resistant to the therapy based on induction of apoptosis [2]. Thus, new medicines that induce nonapoptotic cell death might be useful to eliminate glioma.

Ferroptosis is a newly established type of programmed necrosis and implicated in multiple pathological processes such as neurodegenerative diseases, carcinogenesis, ischemia reperfusion, brain trauma, and cerebral bleeding [3]. Morphologically, it is featured by presence of mitochondria with condensed mitochondrial membrane densities and decreased size [4]. Biochemically, ferroptosis is characterized with intracellular accumulation of iron which results from imbalance between iron uptake, storage, and export [3]. Intracellular iron level is mainly regulated by transferrin receptor (TFR) which accounts for transporting extracellular iron–TF complex into cells via clathrin-mediated endocytosis, ferritin composed of light chain and heavy chain and responsible for storing iron, and ferroportin (FPN) in charge of iron exportation [5]. Iron contributes to cell death via disrupting membrane integrity by peroxidizing polyunsaturated fatty acid (PUFA) chains of membrane phospholipids. Besides serving as a cofactor for nonheme iron‐containing lipoxygenase to enzymatically catalyze PUFA peroxidation [6], iron could react with H_2_O_2_ via Fenton reaction to generate toxic hydroxyl radicals which have potent capacity to peroxidize PUFA [7]. Induction of ferroptosis could effectively eliminate various types of malignant tumor cells from prostate cancer, colorectal cancer, hepatocellular carcinoma, and even cisplatin-resistant lung cancer cells and glioma stem cells [8, 9]. Thus, ferroptosis inducers might be potential medicine for glioma treatment.

Although H_2_O_2_ contributes to lipid peroxidation via Fenton reaction with iron, it remains elusive of the mechanism accounting for its accumulation during the process of ferroptosis. Endoplasmic reticulum (ER) stress is reported to improve intracellular H_2_O_2_ [10], as well as participate in regulation of multiple programmed cell death modes such as apoptosis, parthanatos, and autophagic cell death [10–12]. When ER stress occurs, three signal pathways including PERK, IRE-1, and ATF6 are activated to regulate cellular function [10–12]. As a signal downstream of ER stress PERK/ATF4 pathway, activating transcription factor 3 (ATF3) that belongs to the ATF/CREB family of basic-leucine zipper transcription factors is found to promote H_2_O_2_ generation by initiating NADPH oxidase 4 (NOX4) transcription and inhibit H_2_O_2_ reduction by repressing catalase transcription [13–16]. Moreover, ATF3 is overexpressed in human gliomas [17]. It not only could inhibit tumorigenesis, epithelial–mesenchymal transition, and tumor cell invasion and migration [18–20] but also is involved in regulating the process of ferroptosis, apoptosis, and necrosis [21–23]. However, it remains elusive whether ATF3 plays a role in regulation of glioma cell ferroptosis via promotion of intracellular H_2_O_2_ accumulation.

**Fig. 1:**
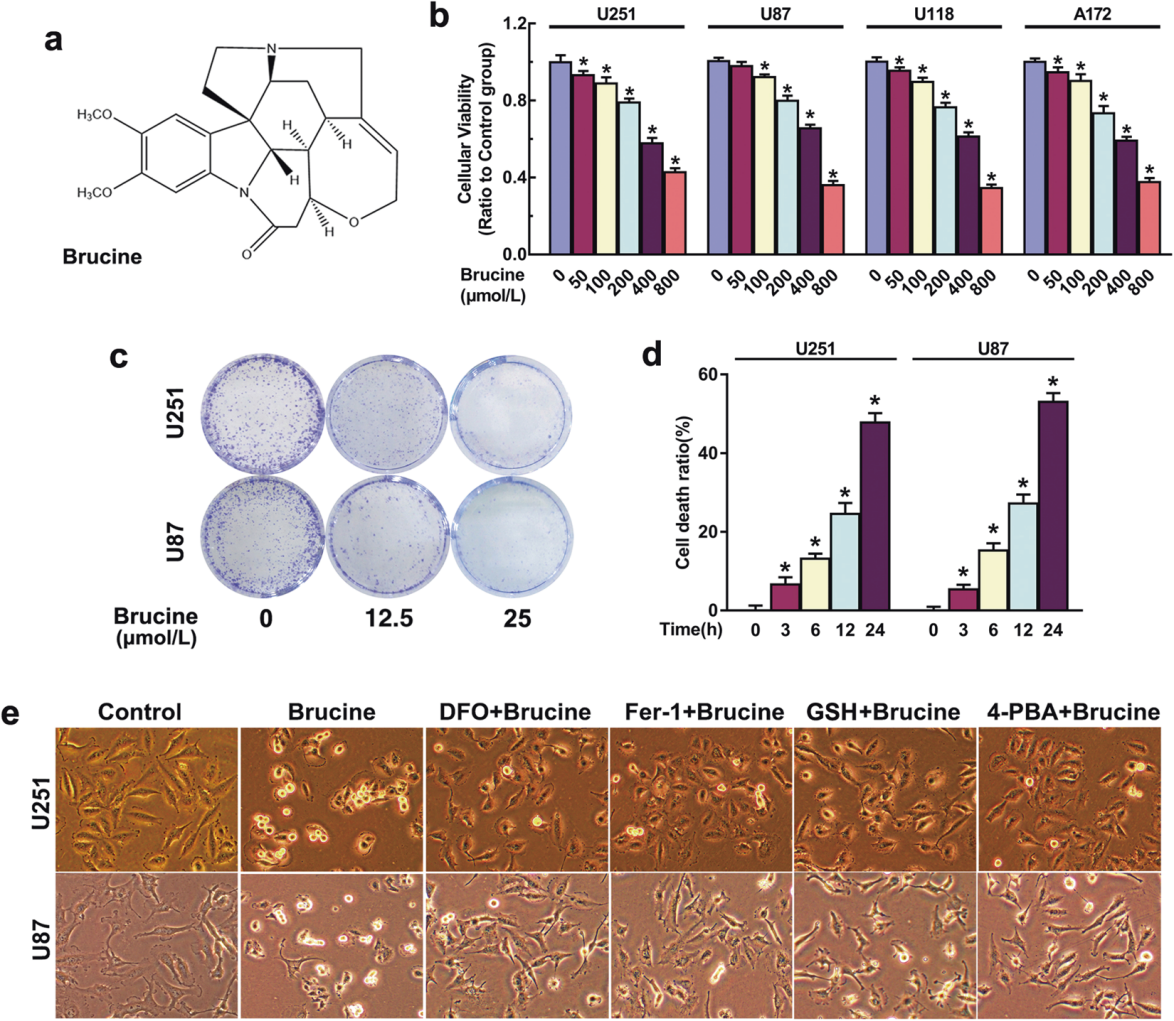
Brucine inhibited glioma cell viability and induced glioma cell death. **a** Chemical structure of brucine. **b** MTT showed that brucine inhibited the viabilities of human U251, U87, U118, and A172 glioma cells in a dosage-dependent manner. **c** Colony formation assay proved that 12.5 μM brucine could obviously inhibit U87 and U251 glioma cells to form colonies, which became more apparent when brucine dosage was increased to 25 μM. **d** LDH release assay showed that brucine at 500 μM induced glioma cell death in a time-dependent manner. **e** Representative images under microscope proved that the cells treated with brucine became shrunk and round when compared with control cells, which was apparently inhibited in the presence of deferoxamine (DFO), ferrostatin-1 (Fer-1), GSH, or 4-PBA. *P < 0.01 versus control group. The values are expressed as mean ± SEM (*n* = 5 per group).

Brucine (Fig. 1a) is a weak alkaline indole alkaloid extracted from the seeds of *Strychnos nux-vomica* and usually used to relieve arthritis and traumatic pain [24]. Recent studies reveal that brucine has potent antitumor activity in various types of cancers, including colon adenocarcinoma, hepatocellular carcinoma, multiple myeloma, breast cancer, and glioma [25–29]. Its antitumor effects are found to be associated with induction of apoptosis, activation of JNK, inhibition of angiogenesis, epithelial–mesenchymal transition, and repression of tumor cell migration and metastasis [25–28, 30]. However, it remains elusive whether ferroptosis and ATF3 are both involved in brucine-induced cancer cell death. Therefore, in this study, we investigated the roles of ferroptosis and ATF3 in brucine-induced glioma cell death and its underlying mechanism.

## Materials and methods

### Reagents

Brucine, ferric ammonium citrate (FAC), and glutathione (GSH) were all purchased from Sigma-Aldrich (Saint Louis, MO, USA). Ferrostatin-1 (Fer-1), liproxastin-1, and 4-phenylbutyrate (4-PBA) were all obtained from Selleck Chemicals (Houston, TX, USA). Primary antibodies against GRP78 (ab12685), ATF3 (ab254268), ATF4 (ab184909), GPX4 (ab125066), cystine-glutamate antiporter xCT (ab175186), ferritin light chain (ab69090), ferritin heavy chain (ab75972), FPN (ab78066), TF (ab82411), TFR (ab1086), ATG5 (ab108327), LC3B (ab192890), p62 (ab109012), Beclin-1 (ab207612), superoxide dismutase 1 (SOD1) (ab51254), and catalase (ab209211) were all from Abcam (Cambridge, UK). Anti-PERK (#5683) and anti-β-Actin (#4970) antibodies were from Cell Signaling Technology (Beverly, MA, USA). The other reagents were purchased from Sigma-Aldrich.

### Cell lines and culture

Human glioblastoma lines (U118, U87, U251, and A172) were obtained from Shanghai Institute of Biochemistry and Cell Biology, Chinese Academy of Sciences (Shanghai, China). The cells were cultured in Dulbecco’s modified Eagle’s medium (DMEM)/high glucose supplemented with 10% fetal bovine serum, penicillin (100 U/mL), and streptomycin (100 μg/mL), and maintained at 37 °C and 5% CO_2_ in a humid environment.

### Assays of cell viability, colony formation, and cell death

Cell viability was examined by using MTT assay kit and was expressed as a ratio of the absorbance at 570 nm to that in control cells.

For colony formation assay, U251 and U87 cells (1 × 10^3^ cells/well) were seeded onto a six-well microplate and cultured for 24 h. After being incubated with brucine at indicated dosages for 14 days, the cells were fixed with 75% ethanol for 30 min and stained with 0.2% crystal violet for 20 min. Medium was changed every 3 days. Colonies of more than 50 cells were counted under microscope.

Cell death was evaluated by using lactate dehydrogenase (LDH) cytotoxicity assay kit from Beyotime Biotechnology (Nanjing, China). According to the manufacturer’s instructions, the absorbance of each prepared sample at 490 nm was read and cell death ratio was calculated using the following formula: Cell death (%) = (Absorbance_sample_ − Absorbance_control_/Absorbance_maximum_ − Absorbance_control_) × 100, where Absorbance_maximum_ is the absorbance of the positive group.

### Measurement of intracellular iron, GSH, and cysteine

Intracellular iron level was determined using an iron colorimetric assay kit from Biovision (Milpitas, CA, USA). In brief, collected cells and glioma tissues (10 mg) were disrupted and homogenized in precooling iron assay buffer and centrifuged at 16,000 × *g* for 10 min at 4 °C to collect the supernatant for testing. The sample was incubated with an equal volume of assay buffer in a 96-well plate for 30 min at room temperature. Then, the sample was incubated with 100 μL of an iron probe in the dark for 60 min at 25 °C, and the absorbance was measured at 593 nm. The iron concentration was calculated using a standard concentration curve. Results are expressed as a ratio to the concentration in control cells.

GSH was measured using a kit from Beyotime Biotechnology (Nanjing, China) as described by the manufacturer. GSH content was expressed as a ratio of the absorbance of each prepared sample at 412 nm to that of control cells.

Cysteine was measured by a kit from Nanjing Jiancheng Bioengineering Institute (Nanjing, China) according to the manufacturer’s instructions. Cysteine content was expressed as a ratio of the absorbance of each prepared sample at 600 nm to that of control cells.

### Measurement of intracellular H_2_O_2_, superoxide anion, and malondialdehyde (MDA)

Intracellular H_2_O_2_ was assayed with a kit from Beyotime Biotechnology (Nanjing, China). According to the manufacturer’s instructions, the absorbance value of each sample was calibrated to a standard concentration curve to calculate the H_2_O_2_ concentration. Results are expressed as a ratio to the concentration of control cells.

Superoxide anion was detected by probe dihydroethidium (DHE) purchased from Beyotime Biotech (Nanjing, China). The cells were cultured in 96-well plates. After being treated with brucine, each group was washed twice in PBS and then loaded with DHE (10 μM) in fresh DMEM at 37 °C in dark for 30 min. After the cells were washed with PBS twice, the fluorescence was measured at an excitation wavelength of 485 nm and an emission wavelength of 530 nm. The levels of superoxide anions were expressed as a ratio to the absorbance value of control cells. The images of U251 and U87 cells stained with DHE as described above were observed by fluorescence microscope (Olympus X71, Tokyo, Japan).

MDA was assayed with a kit from Nanjing Jiancheng Bioengineering Institute (Nanjing, China) according to the manufacturer’s protocols. MDA content was expressed as a ratio of the absorbance of each prepared sample at 532 nm to that of control cells.

### NADPH oxidase activity assay

The activity of NADPH oxidase was determined using a chemiluminescence assay kit purchased from Genmed Scientifics Inc. (Arlington, MA, USA) according to the manufacturer’s instruction. In brief, cells were collected in reagent A and centrifuged at 800 × *g* for 5 min at 4 °C. The supernatant was removed and the pelleted cells were suspended and homogenized in ice-cold reagent B. The samples were vortex for 15 s and incubated on ice for 30 min, then centrifuged at 16000 × *g* for 5 min at 4 °C to obtain the supernatant. The pellet was suspended in reagent B and further homogenized. After protein concentration was measured, 100 μL sample containing 50 μL supernatant and 50 μL pellet was incubated with the test solution (a mixture of reagent C, reagent D, and reagent F) for 2 min at 30 °C, and measured immediately with a luminometer to get the relative light unit (RLU). Finally, the results were expressed as a ratio to RLU of the control cells.

### Transfection of small interfering RNA

siRNA transfection was done using Lipofectamine 3000 (Invitrogen) according to the manufacturer’s instructions with minor modifications. siRNAs were purchased from GenePharma (Suzhou, China): 5′-CCUCAGCAUCUGUUCUUAATT-3′ for NOX4; 5′-GAUGAGAGAAACCUCUUUATT-3′ for ATF3; and 5′-UUCUCCGAACGUGUCACGUTT-3′ for scrambled siRNA (negative control).

### Human U87 tumor xenograft in mice

The athymic BALB/c nude mice (4 weeks; 20–22 g; Beijing Vital River Laboratory Animal Technology Company, China) were housed in a specific pathogen-free environment under a 12-h light–dark cycle with free access to food and water. The study was approved by the Ethics Committee of the First Hospital of Jilin University (Changchun, China). The animals were allowed to acclimatize to their surroundings for 3 days. U87 cells (1 × 10^6^) in the logarithmic growth phase in 100 μL PBS were subcutaneously injected into the right flank. Therapeutic experiments were started when the tumor reached around 150 mm^3^ after about 10 days. Mice were allocated to receive intraperitoneal injections of vehicle (control group, *n *= 6) or 40 mg/kg bodyweight (*n* = 6) in the same volume (50 μL) once a day for 13 times. Tumor size was measured using a slide caliper and tumor volume was calculated using the formula: tumor volume = 0.5 × *A* × *B*^2^ in which *A* is the length, and *B* is the width of the tumor. Tumor tissues were removed after the animals were euthanized by cervical dislocation at the day following final treatment. Tumor tissues were frozen immediately in liquid nitrogen for subsequent assays.

### Gel electrophoresis and Western blotting

The collected glioma cells by centrifugation and the frozen xenografted glioma tissue were homogenized with a glass Pyrex microhomogenizer (20 strokes) in ice-cold lysis buffer (Beyotime Biotech, Nanjing, China). Homogenates were centrifuged at 1000 × *g* for 10 min at 4 °C to obtain supernatant and the pellet. The supernatant was cytoplasmic fraction and the pellet was nuclear fraction. The protein content was determined using Bio-Rad protein assay kit. After SDS electrophoresis and transfer to PVDF membranes, the membranes were blocked with 3% BSA in TBS for 30 min at room temperature, and then incubated overnight at 4 °C with primary antibodies. After incubation with horseradish peroxidase-conjugated secondary antibody and washing the blots, immunoreactive proteins were visualized on a chemiluminescence developer (ChemiScope 5300, Clinx Science Instrument Company, Shanghai, China) and then the density was quantified by using software of ImageJ. The procedure was performed by a researcher who was blinded to group allocation.

### Immunocytochemical staining

The cells seeded on a culture dish were fixed in ethanol, washed with PBS, and incubated with 1% Triton X-100 for 10 min. After the nonspecific antibody binding sites were blocked, the cells were incubated with anti-ATF3 (1:100) followed by incubation in Alexa Fluor 488-conjugated goat anti-rabbit IgG (1:200) for 1 h and then with Hoechst 33258. Finally, all the cells were visualized under laser scanning confocal microscope (Olympus FV1000, Tokyo, Japan) by a researcher who was blinded to group allocation.

### Statistical analysis

Data were obtained from at least four independent experiments. Results are the mean ± SD. Statistical comparisons were made using one-way ANOVA. *P* values less than 0.05 were considered to represent statistical significance.

## Results

### Brucine induced glioma cell death

To investigate the toxic effect of brucine on glioma cells, MTT assay was used to examine the viabilities of U251, U87, U118, and A172 glioma cells in the presence and absence of brucine. After being incubated with brucine at indicated dosages for 24 h, cellular viabilities decreased obviously with the improvement of brucine dosage (Fig. 1b). Then, we examined the long-term toxic effect of brucine on glioma cell proliferation by using colony formation assay. Although U87 and U251 cells were found to form many colonies at 7 days, they were obviously inhibited in the presence of 12.5 μM brucine and further inhibited by 25 μM brucine (Fig. 1c). These indicated that brucine inhibited glioma cells’ viabilities in a dosage-dependent manner. On the basis of the data from MTT assay, we calculated the IC_50_ value of brucine at 24 h and found that it was 604.4 μM in U87 cells, 614.4 μM in U251 cells, 535.9 μM in U118 cells, and 526.8 μM in A172 cells. Thus, we used brucine at 500 μM to test whether brucine could induce glioma cell death. As revealed by LDH release assay, glioma cell death ratio was improved significantly by 500 μM brucine after 3-h incubation, which became more apparent when incubation time was extended to 6, 12, and 24 h (Fig. 1d). Consistently, microscopy revealed that majority of the glioma cells treated with 500 μM brucine became round morphologically at 24 h (Fig. 1e). These indicated that brucine induced glioma cell death in a time-dependent manner. Therefore, brucine not only inhibited the viabilities of glioma cells but also triggered glioma cell death.

### Brucine induced ferroptosis in glioma cells

**Fig. 2:**
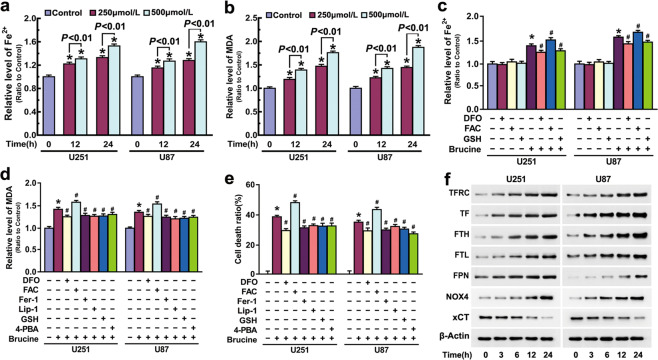
Brucine induced ferroptosis in glioma cells. **a** Iron assay showed that brucine improved intracellular ferrous iron in a time- and dosage-dependent manner. **b** MDA assay proved that brucine induced lipid peroxidation in a time- and dosage-dependent manner. **c** Iron assay revealed that brucine-induced increase of ferrous iron was inhibited by deferoxamine (DFO) and GSH, but reinforced by ferric ammonium citrate (FAC). **d** MDA assay demonstrated that brucine-induced lipid peroxidation was inhibited by deferoxamine (DFO), ferrostatin-1 (Fer-1), liproxstatin-1 (Lip-1), GSH, and 4-PBA, but aggravated by ferric ammonium citrate (FAC). **e** LDH release assay showed that brucine-induced glioma cell death was significantly inhibited by deferoxamine (DFO), ferrostatin-1 (Fer-1), liproxstatin-1 (Lip-1), GSH, and 4-PBA, but exacerbated by ferric ammonium citrate (FAC). **f** Western blotting analysis revealed that brucine triggered time-dependent upregulation of transferrin receptor (TFR), transferrin (TF), ferritin heavy chain (FTH), ferritin light chain (FTL), and NOX4 and downregulation of xCT. *P < 0.01 versus control group. ^#^*P* < 0.01 versus brucine-treated group. The values are expressed as mean ± SEM (*n* = 5 per group).

To elucidate the mechanism accounting for brucine-triggered glioma cell death, we examined brucine-induced changes of ferrous iron and lipid peroxidation. In comparison with control cells, intracellular ferrous iron and lipid peroxidized product MDA were both significantly increased by 250 μM brucine at incubation 12 h, and further improved when incubation time was extended to 24 h or brucine dosage was increased to 500 μM (Fig. 2a, b). These indicated that brucine improved ferrous iron and lipid peroxidation in glioma cells in a dosage- and time-dependent manner.

Thus, U87 and U251 cells were treated for 1 h with iron chelator deferoxamine (DFO) or FAC prior to being incubated with brucine for 24 h. It was found that brucine-induced increase of ferrous iron could be significantly chelated by 500 μM DFO, but reinforced by 500 μM FAC (Fig. 2c). Then, we found that brucine-induced glioma cell death and generation of MDA were both obviously inhibited by DFO or aggravated in the presence of FAC (Fig. 2d). This indicated brucine induced ferrous iron-dependent lipid peroxidation and glioma cell death. To clarify the role of lipid peroxidation in brucine-induced glioma cell death, the cells were treated for 1 h with lipophilic antioxidants Fer-1 at 50 μM and liproxstatin-1 (Lip-1) at 30 μM and then incubated with brucine. We found that MDA, which is a final product of lipid peroxidation and glioma cell death caused by brucine, were obviously inhibited by either Fer-1 or Lip-1 (Fig. 2d, e). Consistently, microscopy revealed that brucine-induced morphological changes in glioma cells were apparently inhibited by DFO and Fer-1 (Fig. 1e). This indicated brucine induced glioma cell death via causing iron-dependent lipid peroxidation. Thus, brucine induced ferroptosis in glioma cells.

To address how brucine improved intracellular iron, Western blotting was used to assay brucine-induced changes in the proteins which could regulate intracellular iron level. It was found that TFR accounting for transporting extracellular TF–iron complex into cells was upregulated by brucine in a time-dependent manner. Accordingly, intracellular level of TF was also markedly improved by brucine at each indicated time. Moreover, ferritin (FTH, FTL), which could bind with intracellular ferrous iron and FPN that could export intracellular iron, was also found to be upregulated by brucine. This indicated that brucine triggered improvement of intracellular iron by promoting iron importation, not via inhibition of ferritin or FPN.

### ATF3 contributed to brucine-induced glioma cell ferroptosis

**Fig. 3:**
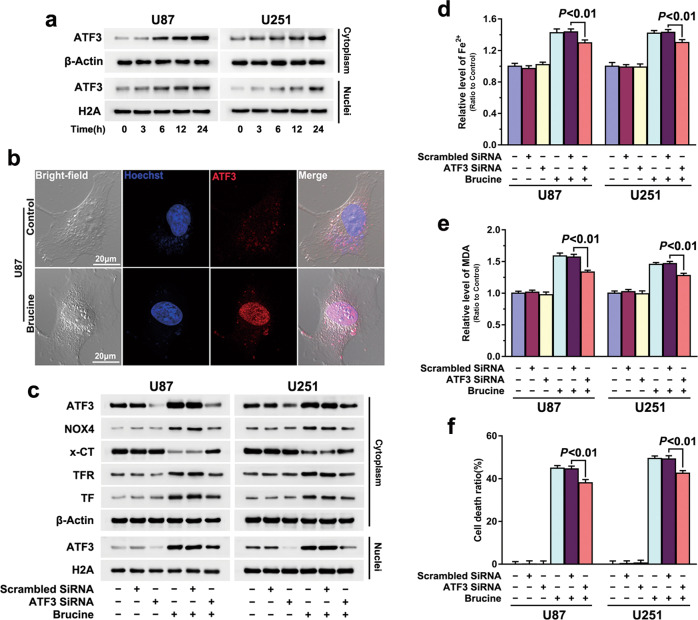
ATF3 contributed to brucine-induced glioma cell ferroptosis. **a** Western blotting analysis showed that brucine treatment resulted in time-dependent upregulation of ATF3 in both cytoplasmic and nuclear fractions in U87 and U251 glioma cells. **b** Representative images of confocal microscopy combined with immune-cytochemistry staining confirmed that ATF3 accumulated apparently in nucleus of the U87 cell treated with brucine for 24 h. **c** Western blotting revealed that knockdown of ATF3 with siRNA prevented brucine-triggered upregulation of NOX4, transferrin, and transferrin receptor and downregulation of xCT. **d** Iron assay proved that ATF3 knockdown prevented iron increase caused by brucine. **e** MDA assay showed that ATF3 knockdown inhibited brucine-induced lipid peroxidation. **f** LDH release assay demonstrated that brucine-induced glioma cell death was significantly prevented when ATF3 was knocked down with siRNA. The values are expressed as mean ± SEM (*n* = 5 per group)

To clarify the factor accounting for brucine-induced glioma cell ferroptosis, we isolated cytoplasmic and nuclear fractions and used Western blotting to analyze brucine-induced changes in ATF3, given that ATF3 plays a crucial role in regulating cell demise [21]. As shown in Fig. 3a, treatment with brucine at 500 μM not only upregulated ATF3 expression but also promoted its translocation into nuclei time-dependently. Confocal microcopy in combination with immune-cytochemistry confirmed as well that ATF3 accumulated obviously at 24 h in the nucleus of the U87 cell treated with brucine, when compared with that in control cell (Fig. 3b). Then, we introduced siRNA to knock down ATF3 and examined its effect on brucine-induced iron increase, lipid peroxidation, and glioma cell death. In comparison with the cells transfected with scrambled siRNA, the upregulated ATF3 triggered by brucine in cytoplasmic and nuclear fractions were both apparently inhibited in the cells transfected with siRNA ATF3 (Fig. 3c). Moreover, brucine-induced increases of ferrous iron and MDA were both inhibited markedly when ATF3 was knocked down with siRNA (Fig. 3d, e). Accordingly, LDH release assay proved that knockdown of ATF3 significantly prevented brucine-induced glioma cell death (Fig. 3f). Thus, ATF3 contributed to brucine-induced glioma cell death by reinforcing iron-dependent lipid peroxidation.

### ATF3 contributed to brucine-induced iron increase by improving H_2_O_2_

**Fig. 4:**
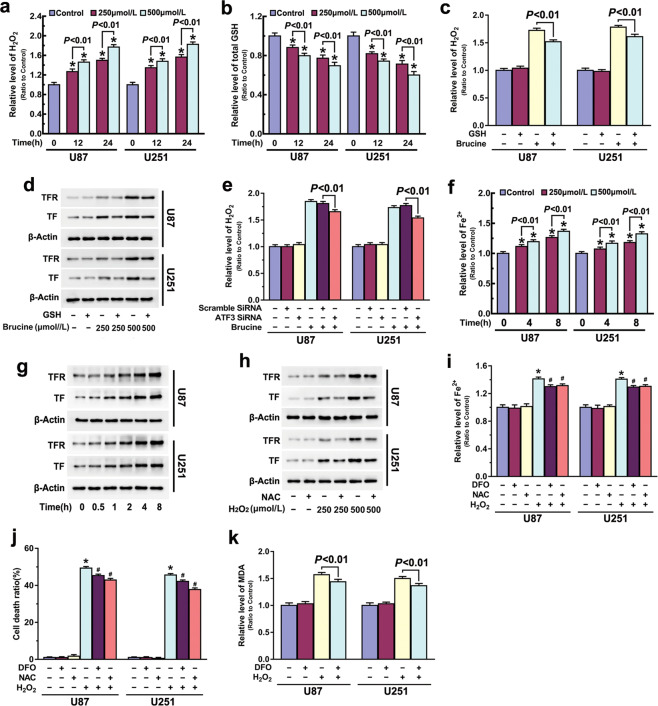
ATF3 contributed to brucine-induced H_2_O_2_ increase. **a** Brucine induced accumulation of H_2_O_2_ in a time- and dosage-dependent manner. **b** Brucine treatment resulted in depletion of GSH in a time- and dosage-dependent manner. **c** Supplement of GSH prevented brucine-induced accumulation of H_2_O_2_. **d** Western blotting revealed that brucine-induced increases of transferrin (TF) and transferrin receptor (TFR) were both inhibited when GSH was supplemented. **e** Knockdown of ATF3 with siRNA prevented brucine-induced accumulation of H_2_O_2_. **f** Iron assay showed that ferrous iron was improved in the cells treated with H_2_O_2_ alone in a time- and dosage-dependent manner. **g** Western blotting revealed that H_2_O_2_ induced time-dependent upregulation of transferrin receptor and transferrin in U87 and U251 glioma cells. **h** Western blotting showed that H_2_O_2_-induced upregulation of transferrin receptor and transferrin was obviously inhibited in the presence of antioxidant NAC. **i** Iron assay showed that H_2_O_2_ induced apparent improvement of ferrous iron, which was inhibited in the cells pretreated with deferoxamine (DFO) and NAC. **j** LDH release assay demonstrated that H_2_O_2_-induced glioma cell death was apparently inhibited by deferoxamine (DFO) or NAC. **k** MDA assay demonstrated that H_2_O_2_ alone could induce lipid peroxidation, which was suppressed in the presence of deferoxamine (DFO). *P < 0.01 versus control group. The values are expressed as mean ± SEM (*n* = 5 per group).

To address whether ATF3 promoted brucine-induced iron increase by improving H_2_O_2_, we examined brucine-induced changes in H_2_O_2_ and the role of H_2_O_2_ in regulation of iron increase. As shown in Fig. 4a, brucine improved intracellular H_2_O_2_ in a time-and dosage-dependent manner, which was paralleled by depletion of intracellular antioxidant GSH at each indicated dosage or incubation time (Fig. 4b). In contrast, both LDH release assay and microscopic observation proved that supplement of exogenous GSH at 5 mM for 1 h significantly inhibited brucine-induced glioma cell death (Figs. 1e and 2e). Then, we found that GSH not only markedly mitigated brucine-induced H_2_O_2_ increase (Fig. 4c) but also suppressed upregulation of TFR and TF and improvement of iron (Figs. 2c and 4d). These indicated that H_2_O_2_ contributed to brucine-induced glioma cell death via improving iron level. Notably, brucine-induced H_2_O_2_ increase was obviously prevented when ATF3 was knocked down with siRNA (Fig. 4e). This indicated that ATF3 promoted brucine-induced iron increase via improving H_2_O_2_.

To further verify the role of H_2_O_2_ in regulation of iron increase in glioma cells, U87 and U251 cells were treated with H_2_O_2_, which could penetrate into cells freely. As shown in Fig. 4f, intracellular ferrous iron was significantly increased at 4 h after the cells were treated with 250 μM H_2_O_2_, and the iron increase became more obvious when incubation time was extended to 8 h or H_2_O_2_ dosage was increased to 500 μM. Consistently, Western blotting proved as well that TFR and TF were both upregulated with incubation time extension from 0.5 to 8 h (Fig. 4g). Moreover, regulatory effect on TF and TFR levels caused by 500 μM H_2_O_2_ was more apparent than 250 μM H_2_O_2_ did (Fig. 4h). In contrast, prior administration of antioxidant NAC at 5 mM for 1 h markedly inhibited H_2_O_2_-induced upregulation of TF and TFR and increase of ferrous iron (Fig. 4h, i). Correspondingly, the glioma cell death caused by H_2_O_2_ was also prevented in the presence of NAC (Fig. 4j). These indicated H_2_O_2_ alone could induce time- and dosage-dependent increase of iron and upregulation of TFR and TF. Then, we found pretreatment with iron chelator DFO at 500 μM for 1 h not only prevented H_2_O_2_-induced increase of ferrous iron (Fig. 4i) but also inhibited lipid peroxidation and glioma cell death (Fig. 4j, k). This indicated that the increased iron contribute to H_2_O_2_-induced lipid peroxidation and glioma cell death. Therefore, H_2_O_2_ contributes to glioma cell ferroptosis via two pathways, one is to increase iron and the other is to cause lipid peroxidation via Fenton reaction with iron.

### ATF3 improved H_2_O_2_ by upregulating NOX4 and SOD1 and downregulating xCT and catalase

**Fig. 5:**
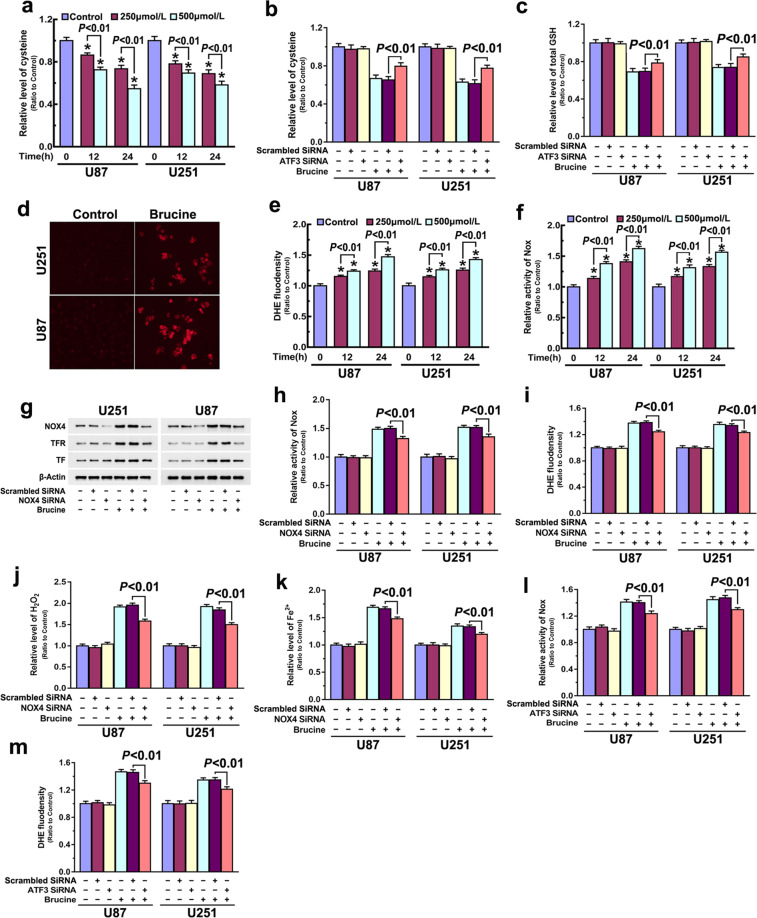
ATF3 regulated brucine-induced NOX4 upregulation and xCT downregulation. **a** Brucine induced time- and dosage-dependent depletion of cysteine in glioma cells. **b** Knockdown of ATF3 with siRNA prevented brucine-induced depletion of cysteine. **c** ATF3 knockdown inhibited brucine-induced GSH depletion. **d** Representative images of fluorescence microscopy showed that the red fluorescence exhibited by DHE was much stronger in brucine-treated cells when compared with that in control cells. **e** Statistical analysis of the fluorescence intensity proved that brucine improved superoxide in a time- and dosage-dependent manner. **f** NADPH oxidase activity assay showed that brucine activated NADPH oxidase in a time- and dosage-dependent manner. **g** Western blotting analysis proved that knockdown of NOX4 with siRNA prevented brucine-triggered upregulation of transferrin (TF) and transferrin receptor (TFR). **h** Brucine-induced upregulation in the activities of NADPH oxidases was suppressed when NOX4 was knocked down. **i** Knockdown of NOX4 prevented brucine-triggered increase of superoxide. **j** Brucine-induced accumulation of H_2_O_2_ was inhibited when NOX4 was knocked down. **k** Knockdown of NOX4 prevented the increase of ferrous iron caused by brucine. **l** Brucine-induced upregulation in NADPH oxidases activities was suppressed when ATF3 was knocked down with siRNA. **m** Knockdown of ATF3 prevented brucine-induced increase of superoxide. The values are expressed as mean ± SEM (*n* = 5 per group).

To clarify the role of ATF3 in promotion of brucine-induced increase of H_2_O_2_, we examined brucine-induced changes in cysteine that could be converted from cystine and then used as a material used for GSH synthesis. As shown in Fig. 5a, cysteine decreased drastically in the U87 and U251 cells treated with brucine at each indicated dosage for indicated time. Western blotting revealed xCT (SLC7A11), which is a specific light-chain subunit of the cystine/glutamate antiporter accounting for transporting extracellular cystine into cells, was time-dependently downregulated by brucine (Fig. 2f). However, GPX4 that could use GSH to reduce lipid oxidation or H_2_O_2_ was upregulated in the presence of brucine (Supplementary Fig. 1a). These indicated that H_2_O_2_ accumulation induced by brucine was associated with downregulation of xCT and catalase. In contrast, knockdown of ATF3 with siRNA not only prevented brucine-induced downregulation of catalase and xCT (Fig. 3c and Supplementary Fig. 1b) but also inhibited depletion of cysteine and GSH (Fig. 5b, c). This suggested that inhibition of xCT and catalase is a pathway via which ATF3 contributed to brucine-induced H_2_O_2_.

Then, we used DHE, a red fluorescence probe of superoxide, to test whether brucine triggered generation of superoxide that could be converted to H_2_O_2_. As revealed by fluorescence microcopy, DHE exhibited much stronger fluorescence in the U87 and U251 cells treated with 500 μM brucine than in control cells (Fig. 5d). Statistical analysis of fluorescence intensity showed that brucine increased superoxide level in a time- and dosage-dependent manner (Fig. 5e). Thus, we assayed the effect of brucine on the activity of NADPH oxidases that could generate superoxide. It was found that the activities of NADPH oxidases were also upregulated with the increase of brucine dosage and the extension of incubation time (Fig. 5f). Then, we used Western blotting to analyze brucine-induced changes in the protein level of NOX4, which is reported to be overexpressed in gliomas [31]. As shown in Fig. 2f, NOX4 was time-dependently upregulated by brucine in glioma cells. Moreover, brucine treatment also resulted in upregulation of SOD1 that could convert superoxide into H_2_O_2_ (Supplementary Fig. 1a).

To verify the role of NOX4 in regulating brucine-induced superoxide and H_2_O_2_, siRNA was introduced to knock down NOX4. It was found that brucine-induced upregulation of NOX4 was obviously inhibited in the cells transfected with NOX4 siRNA when compared with that transfected with scrambled siRNA (Fig. 5g). Moreover, NOX4 knockdown not only suppressed brucine-upregulated activities of NADPH oxidases (Fig. 5h) but also inhibited the increases of superoxide and H_2_O_2_ (Fig. 5i, j). These indicated that brucine induced superoxide generation and H_2_O_2_ improvement via activation of NOX4. Moreover, brucine-induced upregulation of TF and TFR and increase of iron were all inhibited when NOX4 was knocked down (Fig. 5g, k). This further verified that H_2_O_2_ promotes brucine-induced iron increase in glioma cells. Notably, we found that knockdown of ATF3 with siRNA obviously prevented brucine-induced overexpression of NOX4 and SOD1 (Fig. 3c and Supplementary Fig. 1b), NADPH oxidases activation, and superoxide generation (Fig. 5l, m). These indicated that ATF3 also contributed to brucine-induced H_2_O_2_ via upregulation of NOX4 and SOD1.

### Brucine induced a positive feedback between ER stress and H_2_O_2_ generation

**Fig. 6:**
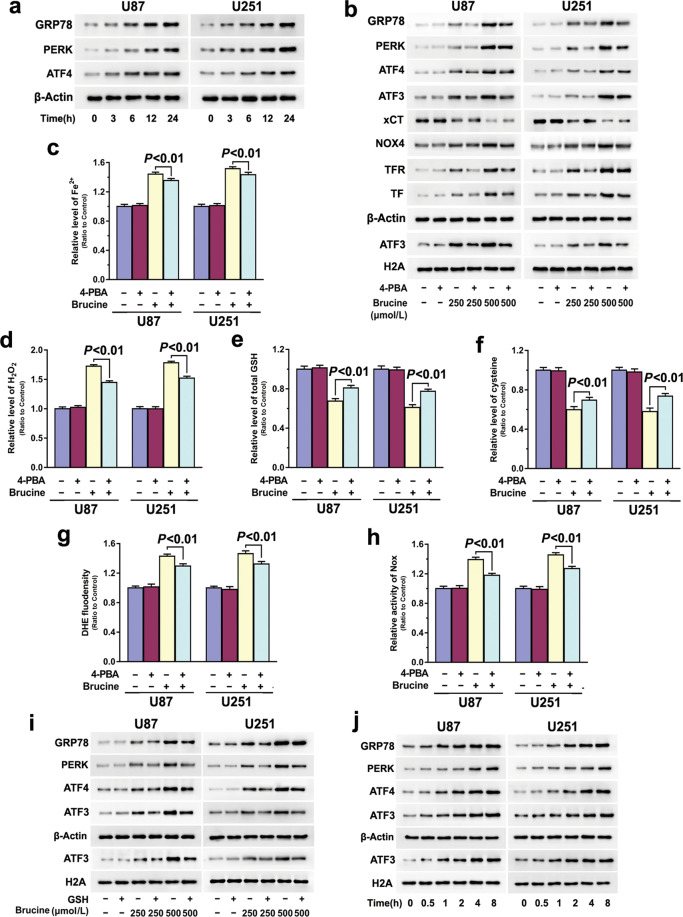
Brucine induced a positive feedback between ER stress and H_2_O_2_ generation. **a** Western blotting showed that brucine upregulated GRP78, PERK, and ATF4 in glioma cells in a time-dependent manner. **b** Western blotting revealed that pretreatment with 4-PBA obviously inhibited brucine-induced upregulation of GRP78, PERK, ATF4, NOX4, transferrin (TF) and transferrin receptor (TFR), downregulation of xCT, and nuclear translocation of ATF3. **c** Iron assay demonstrated that brucine-induced increase of ferrous iron was significantly inhibited by 4-PBA. **d** 4-PBA prevented brucine-induced accumulation of H_2_O_2_. **e** Brucine-induced GSH depletion was suppressed in the presence of 4-PBA. **f** The depletion of cysteine triggered by brucine was prevented in the cells pretreated with 4-PBA. **g** Brucine-induced improvement of superoxide was alleviated by pretreatment with 4-PBA. **h** 4-PBA obviously inhibited brucine-induced activation of NADPH oxidases. **i** Western blotting revealed that 4-PBA prevented brucine-induced upregulation of GRP78, PERK, and ATF4, as well as nuclear translocation of ATF3. **j** H_2_O_2_ could induce upregulation of GRP78, PERK, and ATF4 and nuclear translocation of ATF3. The values are expressed as mean ± SEM (*n* = 5 per group).

To elucidate why brucine could regulate ATF3, we tested whether brucine activated ER stress. As shown by Western blotting, ER stress marker proteins GRP78, PERK, and ATF4 were all time-dependently upregulated by brucine in U87 and U251 cells when compared with those in control cells (Fig. 6a). Moreover, the increases of these marker proteins induced by 500 μM brucine were more apparent than those induced by 250 μM brucine (Fig. 6b). These indicated that brucine activated ER stress in glioma cells in a time- and dosage-dependent manner.

To unveil the role of ER stress in brucine-induced changes in ATF3 and glioma cell ferroptosis, U87 and U251 cells were treated with chemical chaperone 4-PBA at 5 mM for 1 h and then incubated with 500 μM brucine for 24 h. It was found that 4-PBA markedly suppressed the upregulation of GRP78, PERK, and ATF4 caused by brucine at either 250 or 500 μM (Fig. 6b), indicating that 4-PBA effectively suppressed brucine-activated ER stress. Accordingly, LDH release assay and microscopic observation proved that 4-PBA significantly inhibited brucine-induced glioma cell death (Figs. 1e and 2e). Furthermore, we found 4-PBA obviously prevented brucine-induced lipid peroxidation (Fig. 2d), upregulation of TFR and TF, and increases of iron (Fig. 6b, c). These indicated that ER stress contributed to brucine-induced glioma cell ferroptosis. In addition, 4-PBA also apparently inhibited brucine-induced H_2_O_2_ increase (Fig. 6d), depletion of GSH and cysteine and generation of superoxide (Fig. 6e–g), xCT downregulation and NOX4 overexpression (Fig. 6b), and activation of NADPH oxidase (Fig. 6h). These indicated that ER stress contributed to brucine-induced accumulation of H_2_O_2_ via regulation of xCT and NOX4. Notably, brucine-induced upregulation of ATF3 in both cytoplasmic and nuclear fractions was both obviously inhibited by 4-PBA (Fig. 6b), indicating that ER stress contributed to brucine-induced ATF3 upregulation and translocation into nuclei.

In addition, we found that brucine-induced ATF3 upregulation and nuclear translocation, as well as improvement of other ER stress marker proteins GRP78, PERK, and ATF4, were all prevented when H_2_O_2_ was mitigated by GSH (Figs. 4c and 6i). Moreover, Western blotting revealed that GRP78, ATF4, and ATF3 were all time-dependently upregulated in the glioma cells treated with 500 μM H_2_O_2_ alone (Fig. 6j). These indicated that H_2_O_2_ could reversely reinforce brucine-induced ATF3 upregulation and nuclear translocation by exacerbation of ER stress. Therefore, brucine induced a positive feedback between ER stress and H_2_O_2_.

### In vivo study

**Fig. 7:**
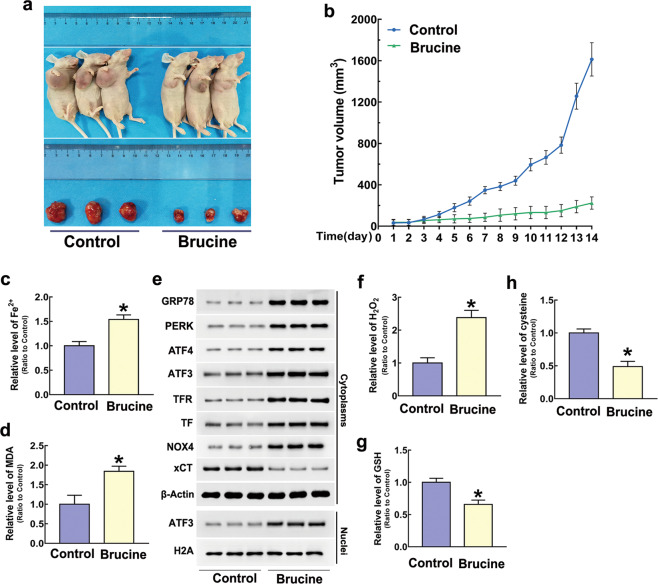
Brucine induced increases of iron and H_2_O_2_ and lipid peroxidation in vivo. **a** Representative images of the nude mice with xenografted gliomas showed that tumor size was significantly smaller in the mice treated with brucine at the dosage of 40 mg/kg for consecutive 13 days than that in control group. **b** Statistical analysis of tumor volumes confirmed as well that TMZ inhibited tumor growth in vivo. **c** Iron assay showed ferrous iron level was significantly higher in brucine-treated group than that in control group. **d** MDA assay proved that lipid peroxidation became more apparent in brucine-treated group when compared with control group. **e** Western blotting analysis revealed that brucine induced marked upregulation of GRP78, PERK, ATF4, NOX4, transferrin (TF), and transferrin receptor (TFR) and downregulation of xCT and nuclear translocation of ATF3. **f** The level of H_2_O_2_ was increased obviously by brucine in vivo. **g** GSH was decreased significantly in brucine-treated group when compared with control group. **h** Cysteine was depleted by cysteine in vivo. *P < 0.01 versus control group. The values are expressed as mean ± SEM (*n *= 6 per group).

To verify the toxicity of brucine in glioma in vivo, U87 cells were xenografted into the right flank of nude mice. Then, we found the tumors in the animals treated with brucine at the dosage of 40 mg/kg for consecutive 13 days were obviously smaller than those in control group (Fig. 7a). Statistical analysis of tumor sizes proved that the growth of brucine-treated tumors was significantly inhibited at day 5 when compared with that not treated with brucine. Although the size of brucine-treated tumors could also increase in the following 9 days, tumor growth was markedly slower than control group (Fig. 7b). Therefore, brucine obviously inhibited glioma cell growth in vivo.

When the treatment was terminated at day 14, the tumors were removed. It was found that the tumor weight was obviously decreased by brucine in comparison with that not treated with brucine (Supplementary Fig. 1c), despite no significant changes could be found in the bodyweight between the mice treated with and without brucine (Supplementary Fig. 1d). Moreover, pathological examination of the heart, lung, liver, spleen, and kidney by using H&E staining showed that brucine at the dose of 40 mg/kg did not produce obvious toxicity to these organs (Supplementary Fig. 1e).

Then, we tested whether brucine treatment could result in improvement of ferrous iron. It was found that ferrous iron and MDA were both significantly improved in brucine-treated tumors in comparison with those of control group (Fig. 7c, d). Western blotting showed as well that TFR and TF were both improved obviously by brucine (Fig. 7e). These indicated that the inhibitory effect of brucine on glioma cells in vivo might be associated with induction of ferroptosis. Moreover, brucine promoted H_2_O_2_ increase, but depleted GSH and cysteine (Fig. 7f, g). Concomitantly, the protein level of NOX4 was upregulated, whereas xCT was downregulated (Fig. 7e). This indicated that brucine improved H_2_O_2_ in glioma cells in vivo via regulation of NOX4 and xCT. Notably, cytoplasmic GRP78 and nuclear translocation of ATF3 were both upregulated in brucine-treated tumors when compared with control ones (Fig. 7e). This indicated that brucine induced ER stress in glioma cells in vivo.

## Discussion

**Fig. 8:**
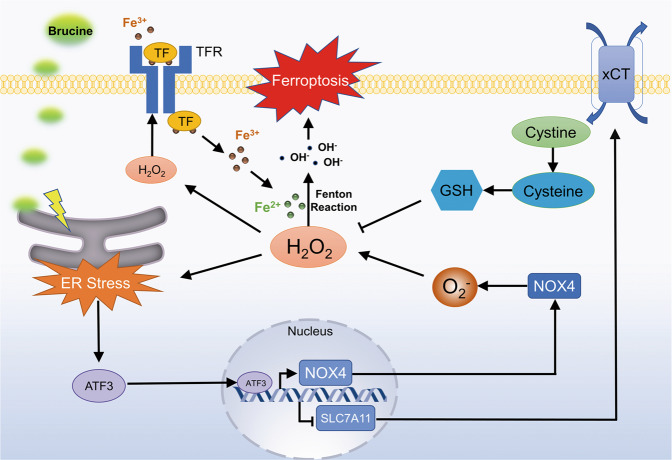
Schematic diagram for the role of ATF3 in brucine-induced glioma cell ferroptosis. Brucine treatment promoted ATF3 upregulation and translocation into nucleus via causing ER stress. Then, ATF3 improved intracellular level of H_2_O_2_ via two pathways. One is to activate NOX4 transcription to excessively generate superoxide, and the other is to repress the transcription of SLC7A11 to deplete cysteine and GSH by inhibition of xCT. The improved H_2_O_2_ not only increased intracellular iron by upregulation of the expression of transferrin receptor, but also reacted with iron via fenton reaction to generate toxic hydroxyl radicals. Eventually, hydroxyl radicals contributed to glioma cell death by causing lipid peroxidation.

In summary, we found in this study that brucine inhibited glioma cell growth in vitro and in vivo, which was paralleled by nuclear translocation of ATF3, increases of iron and H_2_O_2_, and lipid peroxidation. In vitro studies revealed brucine-induced lipid peroxidation was inhibited or exacerbated when intracellular iron was chelated by DFO or improved by FAC. Suppression of lipid peroxidation with Fer-1 or Lip-1 obviously rescued brucine-induced glioma cell death. Thus, brucine induced glioma cell ferroptosis. Notably, knockdown of ATF3 with siRNA significantly inhibited brucine-induced H_2_O_2_ accumulation and iron increase. Mechanistically, ATF3 promoted brucine-induced H_2_O_2_ via upregulating NOX4 and SOD1 to generate H_2_O_2_ and by downregulating xCT and catalase to inhibit H_2_O_2_ reduction. Inhibition of H_2_O_2_ with GSH prevented brucine-triggered iron increase and TFR upregulation. The role of H_2_O_2_ in regulation of iron overload was further verified by treating glioma cells with H_2_O_2_ alone. Moreover, inhibition of brucine-induced ER stress with 4-PBA significantly abrogated ATF3 upregulation and translocation into nuclei. Therefore, ATF3 contributed to brucine-induced ferroptosis via increasing H_2_O_2_ and iron (Fig. 8).

As a form of necrotic cell death, ferroptosis is typically characterized with increase of intracellular iron [8]. The increased iron contributes to cell death via causing excessive lipid peroxidation of polyunsaturated phospholipids on cell membranes [8]. In this study, we found that brucine not only induced glioma cell death but also increased intracellular iron. Moreover, brucine-induced generation of lipid peroxidation product MDA was inhibited or augmented when the increased iron was chelated by DFO or reinforced in the presence of FAC, and suppression of MDA level with Fer-1 or Lip-1 significantly rescued brucine-induced glioma cell death. These are consistent with the biochemical features of ferroptosis reported previously [32]. Different with previous reports showing brucine induced apoptosis in colon adenocarcinoma cells and multiple myeloma cells [25, 27], our data in this study revealed that brucine could trigger ferroptosis in glioma cells.

During the process of ferroptosis, intracellular iron could be improved when TFR is upregulated or ferritin is downregulated. It was reported that genetic knockdown of TFR prevented amino acid starvation-induced ferroptosis in mouse embryonic fibroblasts [33]. Similarly, cardiomyocytes in ferritin-deficient mice were sensitive to ferroptosis induced by higher iron diet in comparison with normal cardiomyocytes [34]. In this study, we found that brucine not only increased intracellular iron but also upregulated the protein level of TFR in a time-dependent manner. Although ferritin light chain and ferritin heavy chain were both upregulated by brucine, we think that it is a secondary response of glioma cells to increased iron. Thus, brucine improved intracellular iron in glioma cells via upregulating TFR.

H_2_O_2_ plays a crucial role in regulation of ferroptosis, given that it not only cause lipid peroxidation via Fenton reaction with iron but also could upregulate the expression of TFR [32]. Notably, the regulatory effect of H_2_O_2_ on TFR expression was found to be associated with activation of autophagy [35, 36]. In this study, we found that brucine not only improved H_2_O_2_ level but also triggered upregulation of autophagy-related marker proteins ATG5, beclin-1 and LC3BII, and downregulation of autophagy substrate p62 (Supplementary Fig. 1a). Thus, autophagy might participate in regulation of brucine-induced upregulation of TFR. Moreover, we also found that mitigation of brucine-induced H_2_O_2_ with GSH obviously inhibited iron increase and TFR upregulation in glioma cells. Furthermore, intracellular iron and TFR were both apparently upregulated when glioma cells were treated with exterior H_2_O_2_. Therefore, H_2_O_2_ contributed to brucine-induced iron increase via upregulation of TFR. In addition, it was also reported that H_2_O_2_ permeability was increased when lipid peroxidation occurred in cancer cell lines [37], suggesting that H_2_O_2_ might be released from ferroptotic cells and attack neighboring cells. Thus, H_2_O_2_ plays a crucial role in ferroptotic cell death.

H_2_O_2_ accumulation within cells is the result of disrupted balance between its generation and clearance [7]. NADPH oxidase and SOD1 could collaborate to improve intracellular H_2_O_2_ generation, because the superoxide produced by NADPH oxidase is converted into H_2_O_2_ in the presence of cytoplasmic SOD1 [38]. It was reported that NADPH oxidase promoted paraquat-induced ferroptosis in dopaminergic neurons and heart failure-triggered ferroptosis in myocardiocytes [39, 40]. Similarly, SOD1 was found to be involved in sepsis-induced oxidative stress and ferroptosis in myocardiocytes [38]. As a member of NADPH oxidase, NOX4 is overexpressed in human gliomas and contributed to pseudolaric acid B-triggered glioma cell ferroptosis via increasing H_2_O_2_ [7]. In this study, we found that brucine not only induced NOX4 activation but also upregulated the protein levels of NOX4 and SOD1 in glioma cells, knockdown of NOX4 with siRNA obviously prevented brucine-induced increases of superoxide and H_2_O_2_. Therefore, brucine improved intracellular H_2_O_2_ via upregulation of NOX4 and SOD1.

Intracellular H_2_O_2_ could be decreased by catalase which can convert H_2_O_2_ reduction into oxygen and water [41]. Moreover, GPX4/GSH is also an intracellular pathway to decrease H_2_O_2_ level, given that GPX4 catalyzes H_2_O_2_ reduction at the expense of GSH [42]. GSH is synthesized from cysteine, and cysteine is converted from cystine that is imported into cells by cystine-glutamate antiporter xCT [43]. Therefore, intracellular H_2_O_2_ could be increased when catalase was downregulation of or GSH was depleted by suppression of xCT. Consistently, it was reported that pharmacological inhibition of xCT with erastin was an effective way to induce ferroptosis in various types of cancer cells [43]. In this study, we found that brucine treatment resulted in depletion of GSH and cysteine and downregulation of xCT and catalase, despite GPX4 was upregulated. In contrast, supplement of GSH significantly inhibited brucine-induced H_2_O_2_ increase. Thus, brucine also improves H_2_O_2_ via downregulation of xCT and catalase.

Accumulating evidences have shown that ATF3 plays a crucial role in improving intracellular H_2_O_2_ as a transcription activator or repressor. It not only promote H_2_O_2_ generation by initiating NOX4 transcription but also inhibit H_2_O_2_ reduction by repressing catalase transcription [14–16], as well as regulate apoptosis and necrosis [23], ATF3 also participates in modulation of ferroptosis. It was reported that ATF3 contributed to erastin-induced ferroptosis in retinal pigment epithelial cells via causing GSH depletion by inhibiting xCT transcription [21]. In this study, we found brucine induced ATF3 upregulation and translocation into nuclei in glioma cells. Knockdown of ATF3 with siRNA not only inhibited brucine-induced H_2_O_2_ increase but also prevented upregulation of NOX4 and SOD1 and downregulation of catalase and xCT. Different with previous study showing that ATF3 aggravated ischemia reperfusion- or folic acid-induced ferroptosis in renal tubular epithelial cells by exacerbation of GPX4 inhibition [44], we found in this study that knockdown of ATF3 inhibited brucine-induced upregulation of GPX4. Therefore, ATF3 contributed to brucine-induced H_2_O_2_ by upregulation of NOX4 and downregulation of catalase and xCT.

ATF3 expression is primarily stimulated by PERK/ATF4-mediated pathway that is activated when ER stress occurs [45]. In this study, we found that ER stress marker proteins GRP78, PERK, and ATF4 were all upregulated in brucine-treated glioma cells. In contrast, brucine-induced ATF3 upregulation and translocation into nuclei was obviously inhibited when the upregulated PERK and ATF4 were alleviated by chemical chaperone 4-PBA. Therefore, PERK/ATF4 pathway might contribute to brucine-induced changes in ATF3. In addition, H_2_O_2_ could activate ER stress, which was supported by that H_2_O_2_ treatment resulted in activation of ER stress in myogenic cells [46]. Similarly, we found in this study that GRP78, PERK, and ATF4, as well as ATF3, were all significantly upregulated in the human glioma cells stressed with H_2_O_2_ alone. Consistently, inhibition of brucine-induced H_2_O_2_ by supplement of GSH obviously prevented the upregulation of GRP78, PERK, ATF4, and ATF3. Therefore, brucine induced a positive feedback between ER stress and H_2_O_2_ accumulation in glioma cells, which regulated mutually to promote glioma cell ferroptosis.

In conclusion, we demonstrate in this study that brucine activated ER stress in glioma cells, which results in ATF3 upregulation and nuclear translocation. Then, ATF3 contributes to intracellular accumulation of H_2_O_2_ by upregulation of NOX4 and downregulation of xCT and catalase. Eventually, H_2_O_2_ leads to glioma cell death via causing TFR-regulated iron overload.

## Supplementary information

Supplementary Information
