# Supplementary figures and images for: ATF3 contributes to brucine-triggered glioma cell ferroptosis via promotion of hydrogen peroxide and iron
Source: Acta Pharmacol Sin. 2021 Jun 10;42(10):1690–702. doi: 10.1038/s41401-021-00700-w (PMC8463534; doi:10.1038/s41401-021-00700-w)

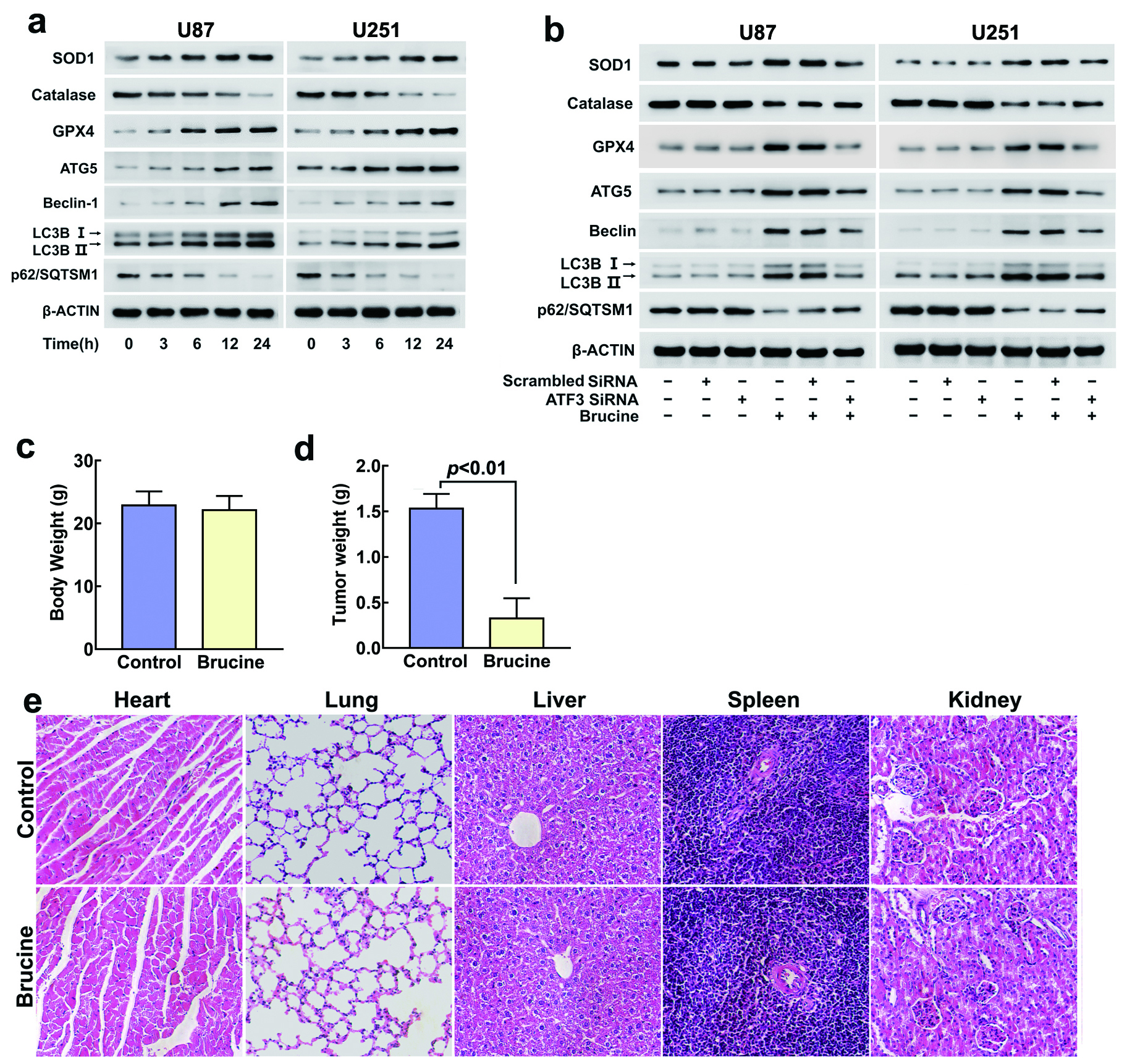

Supplement: Supplementary file 1 — Supplementary Information [file 41401_2021_700_MOESM1_ESM.tif]
